# Supplementary material for: PI3K/mTOR Dual Inhibitor Pictilisib Stably Binds to Site I of Human Serum Albumin as Observed by Computer Simulation, Multispectroscopic, and Microscopic Studies
Source: Molecules. 2022 Aug 9;27(16):5071. doi: 10.3390/molecules27165071 (PMC9413508; doi:10.3390/molecules27165071)
Supplement: Supplementary file 1 [file molecules-27-05071-s001.zip › molecules-1816003-supplementary.pdf]

molecules MDPI

Supplementary Materials

PI3K/mTOR Dual Inhibitor Pictilisib Stably Binds to the Site I of the Human Serum Albumin as observed by Computer Simulation, Multispectroscopic, and Microscopic Studies

Hongqin Yang*,†, Yanjun Ma*,†, Hongjie Zhang, Junyi Ma

College of Life Sciences, Northwest Normal University, Lanzhou 730070, China;
* Correspondence: yanghongqin@nwnu.edu.cn (H.Y.); mayjdyx@139.com (Y.M.)
† These authors contributed equally to this work.

Supplementary Table S1. The decomposition of binding energies (all of the energies analysis was carried out based on simulation time from 75 to 95 ns).

Energy Component	Average (kcal/mol)	Std. Dev.	Std. Err. of Mean
ΔE_{vdw}	-58.4997	2.6891	0.1901
ΔE_{ele}	-13.6927	5.7237	0.4047
ΔG_{GB}	31.8142	4.9574	0.3505
ΔG_{SA}	-6.7683	0.2866	0.0203
ΔG_{bind}	-47.1465	3.7015	0.2617

Supplementary Table S2. Fluorescence decay parameters of HSA (2 μM) in the absence and pres-ence of various concentrations of GDC-0941. τ in ns.

Sample	τ_1	τ_2	τ_3	α_1	α_2	α_3	τ_{avg}	χ^2
0	3.4090	0.4726	7.0099	0.3616	0.0332	0.6052	5.4907	1.0919
3 μM	3.3932	0.4749	6.9171	0.3690	0.0319	0.5991	5.4113	1.0380
6 μM	3.2753	0.4793	6.7937	0.3525	0.0414	0.6061	5.4133	1.1072

Supplementary Table S3. 3D fluorescence spectral parameters of HSA in the absence and presence of GDC-0941.

HSA–GDC-0941	Peak No.	Peak Position [$\lambda_{ex}/\lambda_{em}$ (nm/nm)]	Stokes Shift $\Delta\lambda$(nm)	Intensity
1:0	A	280/280→350/350	-	150.79→224.96
	B	240/480	-	265.34
	I	280/337	57	514.50
	II	230/338	108	545.36
1:1	A	280/280→350/350	-	146.94→223.73
	B	240/480	-	289.99
	I	280/338	58	437.24
	II	230/333	103	402.67
1:3	A	280/280→350/350	-	135.69→211.31
	B	240/480	-	280.66
	I	280/340	60	387.28
	II	230/334	104	239.51
